# Is the reflux disease questionnaire useful for identifying GERD according to the Montreal definition?

**DOI:** 10.1186/1471-230X-14-17

**Published:** 2014-01-22

**Authors:** Enrique Rey, Marta Barceló, Javier Zapardiel, Eduardo Sobreviela, Mercedes Muñoz, Manuel Díaz-Rubio

**Affiliations:** 1 Department of Digestive Diseases, San Carlos Clinical Hospital, Madrid Complutense University, Madrid 28040, Spain; 2 Microbiology, Infanta Elena Hospital, Madrid, Spain; 3 Biometrics Department, Quintiles Iberia, Madrid, Spain; 4 Medical Department, AstraZeneca, Spain

**Keywords:** GERD, Heartburn, Questionnaires, Patient-reported outcomes

## Abstract

**Background:**

Scales for aiding physicians diagnose gastro-oesophageal reflux disease (GERD) have not been evaluated in terms of their ability to discriminate between troublesome symptoms (TS) and non-troublesome symptoms (NTS). Our objective is to evaluate the ability of the Reflux Disease Questionnaire (RDQ) to identify GERD according to referral of TS, in patients without previous proton pump inhibitor (PPI) treatment and in patients on PPI treatment.

**Methods:**

Patients consulting physicians because of heartburn or acid regurgitation were recruited at 926 primary-care centres in Spain. They were asked to complete several questionnaires including the RDQ, and to define which of their symptoms were troublesome. Information on drug treatment was collected by the physician. We performed a receiver operating characteristic (ROC) curve analysis to ascertain the RDQ's optimum cut-point for identifying TS.

**Results:**

4574 patients were included, 1887 without PPI and 2596 on PPI treatment. Among those without PPI treatment, 1722 reported TS. The area under the curve (AUC) was 0.79 for the RDQ, and the optimum RDQ cut-point for identifying TS was 3.18 (sensitivity, 63.2%; specificity, 80.2%). A total of 2367 patients on PPI treatment reported TS, and the optimum RDQ cut-off value was 3.06 (sensitivity, 65.4%; specificity, 71.8%).

**Conclusions:**

An RDQ score higher than 3 shows good sensitivity and specificity for differentiating TS from NTS among patients without PPI or on PPI treatment. The RDQ is useful in primary care for diagnosis of GERD based on the Montreal definition.

## Background

In Western societies, 30% [1] to 60% [2] of persons suffer from heartburn or regurgitation, yet not all those who report these symptoms suffer from gastro-oesophageal reflux disease (GERD). Indeed, the differentiation between frequent and occasional symptoms is unsatisfactory from a practical standpoint, in view of the fact that a considerable proportion of patients with occasional symptoms consult their physician [3] and suffer from a deterioration in their quality of life [4]. Consequently, for every patient that seeks advice, it is the physician who has to decide whether the symptoms are irrelevant or circumstantial (and, by extension, not constitutive of disease), or alternatively, whether the patient is really suffering from GERD.

The Montreal Consensus defines GERD as "a condition which develops when the reflux of stomach contents causes troublesome symptoms and/or complications" [5], the conclusion being that, in clinical practice, it is the patient who defines whether or not the symptoms are troublesome, without the use of arbitrary cut-points of frequency and severity. Any given person's perception of a symptom as troublesome goes beyond its frequency, its severity or even its impact on quality of life [6], since it implies individual cognitive assessment which may vary according to the circumstances [7].

When it comes to the severity of GERD -an aspect of crucial importance to the physician because it is precisely on this that his attitude to diagnosis and therapy will so often depend [8,9]- the Montreal Consensus does not specify how GERD severity is to be assessed in clinical practice, thus assuming the degree of severity to be that reported by the patient [10].

Yet, this commitment to patient-centred clinical practice comes up against the reality of the fact that, as compared to the opinion held by their patients, physicians tend to underestimate the impact of gastro-oesophageal reflux symptoms [11,12]. To try and minimise the discrepancies between patient-based information and physicians' subjective evaluation, useful instruments have been developed to collect information directly from the patient as uniformly as possible and help physicians diagnose GERD and assess its severity, e.g., the Reflux Disease Questionnaire (RDQ) [13-15]. This questionnaire was developed using definitions of GERD based on symptom frequency and severity, and its ability to correctly identify GERD was recently validated using 24-hour pH monitoring as a gold standard [16]. However, GERD is usually diagnosed without invasive testing, and the neither of the following aspects is known: the extent to which the RDQ is useful for differentiating troublesome from non-troublesome reflux symptoms, a key factor for diagnosing GERD within the new conceptual framework proposed by the Montreal Consensus; and, the RDQ's capacity to distinguish symptom severity.

Hence, the primary objective of this study was to assess the utility of the RDQ in standard clinical practice for identifying reflux symptoms defined as troublesome by the patient. As a secondary objective, we proposed to evaluate its capacity for classifying the severity of GERD symptoms.

## Methods

### Design

This was an observational, cross-sectional multicentre study conducted at primary-care centres nationwide. The study was formally approved by the Clinical Research Ethics Committee of the San Carlos Hospital (*Hospital Clínico San Carlos*) in Madrid, and informed consent was obtained from all participants prior to their inclusion. Data-collection was performed from September to November 2007.

### Study population and inclusion process

The study enrolled consecutive patients attending a primary-care facility and presenting with typical GERD symptoms (heartburn and/or acid regurgitation). Any patient aged 18 years or over who reported heartburn or acid regurgitation in the three months preceding the medical visit was defined as eligible, with the following deemed ineligible: patients whose reason for medical consultation was simply to renew their prescription; patients with severe diseases which, in the researcher's opinion, could significantly affect health-related quality of life; and patients unable to read/complete the questionnaires because of mental or physical disability.

Eligible patients completed a screening questionnaire containing three questions, with a recall period of three months. The first two questions referred to whether the patient had suffered heartburn or acid regurgitation. These questions were drawn from the GastroEsophageal Reflux Questionnaire [17], adapted and validated for use in Spanish [18]. The third question in the screening questionnaire was aimed at classifying symptoms as troublesome or non-troublesome, and was worded as follows, "*Would you say that the burning feeling that rises through your chest or the liquid coming back into your mouth leaving a bitter or sour taste has been a troublesome problem*?" This question was intended to act as a uniform reminder to patients for the purpose of reporting troublesome/non-troublesome symptoms and was reworded in Spanish after a group of clinical gastroenterologists agreed that the question was both meaningful and recalled what it meant to recall.

Patients who reported heartburn or acid regurgitation in the first two questions of the screening questionnaire were included in the study. To ensure inclusion of a sufficient number of patients with non-troublesome symptoms, each researcher included a patient with symptoms classified as non-troublesome for every 4 who perceived their symptoms as troublesome.

### Data-collection

Data were drawn from two sources: [1] self-administered questionnaires designed to collect information directly from patients; and [2] clinical information collected directly by the physician (anamnesis and clinical history) on the basis of a pre-established form.

#### *Instruments (patient-reported outcomes)*

Patients completed a number of questionnaires administered to obtain information on reflux symptoms (RDQ), impact of GERD symptoms (GERD impact scale, GIS), digestive symptoms and severity of reflux symptoms (Gastrointestinal Symptom Rating Scale, GSRS), and quality of life (Quality of Life in Reflux and Dyspepsia, QOLRAD).

The RDQ was developed to be used as a diagnostic tool and to monitor treatment response over time. The RDQ evaluates 6 symptoms covering three domains (heartburn, regurgitation and upper abdominal pain) using a 6-point Likert scale to assess frequency and severity within the preceding week. Each answer was rated from 1 to 6, and the RDQ mean score was then calculated as the mean of the respective responses to the 12 items; RDQ mean scores thus ranged from 1 to 6. The RDQ, which has been psychometrically validated [13] and shown its utility for diagnosing GERD [16], has also been adapted and validated in Spanish [14].

While the GIS is a self-administered questionnaire designed to improve patient-doctor communication and furnish information on treatment outcomes, it has not been formally validated as a tool for monitoring the response of GERD symptoms and quality of life to proton pump inhibitor (PPI) treatment [19]. The questionnaire scores the frequency of 9 items and uses a 4-point Likert scale, with a one-week recall period. The GIS score is calculated as the mean value of all item responses, with the scale being scored from 1 to 4, such that the higher the value, the better the patient. It is a management tool [15] and the psychometric validity of the Spanish translation has recently been reported [14].

The GSRS was used to describe subjects' symptom clusters. The GSRS covers 15 gastrointestinal symptoms and uses a 7-point Likert scale to rate each symptom. The GSRS contains five dimensions, which respectively address "Indigestion" (four items), "Diarrhoea" (3 items), "Constipation" (3 items), "Abdominal pain" (3 items) and "Reflux" (2 items) in the preceding week. The GSRS is scored in such a way that the lower the value, the less severe the perceived gastrointestinal symptoms. The Reflux dimension (heartburn and acid regurgitation) was used to assess GERD severity, with a score of less than 3 being deemed mild and a score of 5 or more, severe [20]. Both the reliability and validity of the Spanish version of the GSRS have previously been documented [21].

The impact of upper GI symptoms on quality of life was assessed using the QOLRAD questionnaire. The QOLRAD questionnaire comprises 25 items with a recall period of one week, grouped into the following dimensions: "Emotional distress"; "Sleep dysfunction"; "Vitality"; "Food/drink problems"; and "Physical/social functioning". Questions are rated on a 7-point Likert scale and the global score then calculated, such that the lower the value, the more severe the impact on health-related quality of life. The QOLRAD has been documented as being a reliable, valid and responsive instrument in subjects with heartburn [22].

#### *Clinical data*

Clinical data were recorded directly by the physician in accordance with pre-coded categories. This information referred to: socio-demographic data (age, sex, occupational status, marital status, educational level); anthropometric data (weight in kg, height in cm); alcohol consumption (none, a minimum of once per week); smoking habit (never-smoker, ex-smoker, smoker); associated diseases (diabetes, arterial hypertension, hypercholesterolaemia, anxiety disorder, depression and asthma); consumption of medical drugs (calcium antagonists, nitrates, benzodiazepines, nonsteroidal anti-inflammatory drugs, aspirin); and use of GERD drugs, including antacids, H2-blockers and PPIs (at any dose and under any guideline). In addition, physicians were asked to classify the severity of patients' GERD symptoms as mild, moderate or severe, on the basis of their own judgement.

### Statistical analysis

For analysis purposes, the sample was stratified into two groups, namely, [1] patients off PPI i.e., patients not receiving treatment with PPIs, and [2] patients on PPI, i.e., patients on treatment with PPIs, given that these represent two potentially different clinical scenarios. All analyses were performed separately in both groups, using the same definitions of variables and test statistics.

Continuous variables were expressed as mean ± standard deviation (interval) and categorical variables as absolute and relative frequencies. For comparison purposes, the Student's T-test was used for continuous variables, and the Chi-squared or Fisher's Exact test for qualitative variables. Hypothesis tests were deemed statistically significant at *p* < 0.05.

In line with their responses to the three screening questions, patients were classified into the following two groups: [1] troublesome symptoms (TS), i.e., patients with heartburn and/or regurgitation classified as troublesome; and [2] non-troublesome symptoms (NTS), i.e., those who classified their heartburn and/or regurgitation as non-troublesome.

To assess to what extent factors extraneous to the characteristics of the symptoms influenced the classification of symptoms as TS or NTS, a logistic regression model was constructed, including the impact of symptoms as evaluated by the GIS, age, sex, body mass index (BMI), alcohol consumption and smoking, as well as the presence of comorbidities.

To evaluate the ability to distinguish between troublesome and non-troublesome symptoms in the global RDQ score, as well as the optimal cut-point, the corresponding receiver operating characteristic (ROC) curves were plotted. Based on these, the optimal cut-point was calculated, predefined as that which would maximise Younden's index (S + Sp-1).

ROC curves were also plotted to ascertain cut-points in the RDQ scale capable of classifying GERD symptoms as mild, moderate or severe, with the classification of severity obtained by the GSRS being used as the gold standard.

The degree of agreement between severity of GERD symptoms obtained on the basis of the GSRS, RDQ, and physicians' subjective evaluation of symptom severity was estimated using Kappa's weighted index.

### Sample size

Assuming that, once the optimal point has been chosen, both scales have an S ≥ 70% and an Sp ≥ 70%, and that there would be no differences between patients on and those not on PPI treatment, then, based on a sample of 5621 patients, the sensitivity and specificity of each test could be estimated with a maximum admissible error of δ1 = ±1.35% and δ2 = ±2.60% respectively. Given that the inclusion of one patient with NTS was requested for every 4 with TS, 20% of patients could be assumed to present with non-troublesome GERD symptoms. These estimates were calculated with a 95% two-sided confidence interval.

On the assumption that 5% of the patients included in the study would not be valid for analysis, a total of 5918 patients would have to be included.

## Results

On the basis of the screening criteria, the study initially included a total of 5332 eligible patients attending 926 centres distributed nationwide (48 out of 51 provinces): 758 patients were excluded for not fulfilling the inclusion and exclusion criteria, due to the lack of some item of basic information (e.g., age). Accordingly, 4574 (85.8%) patients were available for analysis; of these, 4410 patients reported heartburn and 4230 acid regurgitation in the screening questions.

### Patients without PPI treatment

Of the 4574 analysable patients, 1968 (43%) were not on treatment with PPIs; 1722 (85.7%) regarded their reflux symptoms as troublesome, and 246 (14.3%) regarded them as non-troublesome. Table 1 shows the differences between patients who reported their symptoms as TS and those who reported them as NTS. As against patients who reported their symptoms as non-troublesome, patients with troublesome symptoms were slightly older, a smaller proportion were women, and a higher proportion consumed no alcohol or consumed it only occasionally, with the sole difference of note being an appreciably higher proportion of persons diagnosed with hypercholesterolemia among patients with TS. All symptoms were more frequent among patients who reported TS. In the logistic regression analysis, the factors associated with reporting TS were, diagnosis of hypercholesterolaemia, frequency of acid regurgitation, epigastric pain/burning (probably reflecting related dyspepsia) and sleep disturbance due to the reflux symptoms, with the pertinent Odds Ratios (95% CI) being shown in Table 2.

**Table 1:** **Characteristics of patients off PPI**, **stratified according to whether symptoms were non**-**troublesome or troublesome**

	**Variable**	**Non**-**troublesome symptoms (N = 246)**	**Troublesome symptoms (N = 1722)**
Age*	Mean ± SD	50.0 ± 12.3	52.0 ± 12.8
Gender*	% females	123 (50%)	739 (42.9%)
Body mass index	Mean ± SD	26.3 ± 3.7	26.9 ± 3.7
Alcohol*	Any	188 (76.3%)	1233 (71.6%)
Smoking	Current	122 (49.9%)	928 (53.9%)
	Former	69 (28%)	408 (23.7%)
Concomitant therapy	Calcium blockers	12 (4.9%)	129 (7.5%)
	Nitrates	3 (1.2%)	17 (1.0%)
	NSAIDs	22 (8.9%)	201 (11.7%)
	ASA	9 (3.6%)	86 (5.0%)
	Benzodiazepines	29 (11.8%)	219 (12.7%)
Comorbidity	Diabetes	17 (6.9%)	141 (8.2%)
	Hypertension	63 (25.6%)	513 (29.8%)
	Hypercholesterolaemia**	36 (14.6%)	410 (23.9%)
	Depression	14 (5.7%)	139 (8.1%)
	Anxiety disorder	53 (21.5%)	387 (22.5%)
	Asthma	9 (3.6%)	77 (4.5%)
Frequency of symptoms+			
Heartburn**	Never	46 (18.8%)	124 (7.2%)
	Sometimes	143 (58.4%)	708 (41.1%)
	Often	48 (19.6%)	699 (40.6%)
	Daily	8 (3.3%)	191 (11.1%)
Acid regurgitation**	Never	65 (26.9%)	123 (7.2%)
	Sometimes	139 (57.4%)	739 (43.1%)
	Often	34 (14.0%)	661 (38.5%)
	Daily	4 (1.7%)	193 (11.2%)
Chest pain**	Never	127 (51.8%)	444 (25.8%)
	Sometimes	93 (38.0%)	767 (44.5%)
	Often	21 (8.6%)	429 (24.9%)
	Daily	4 (1.6%)	82 (4.8%)
Epigastric burning/pain**	Never	66 (26.8%)	184 (10.7%)
	Sometimes	136 (55.3%)	732 (42.5%)
	Often	39 (15.9%)	643 (37.3%)
	Daily	5 (2.0%)	163 (9.5%)
Hoarseness**	Never	157 (64.6%)	582 (33.9%)
	Sometimes	68 (28.0%)	749 (43.6%)
	Often	15 (6.2%)	315 (18.3%)
	Daily	3 (1.2%)	73 (4.2%)
Impact of symptoms+			
Sleep disturbance**	Never	106 (43.3%)	233 (13.5%)
	Sometimes	115 (46.9%)	938 (54.5%)
	Often	24 (9.8%)	478 (27.8%)
	Daily	0 (0.0%)	72 (4.2%)
Eating disturbance**	Never	104 (42.4%)	276 (16.0%)
	Sometimes	116 (47.3%)	890 (51.7%)
	Often	23 (9.4%)	476 (27.6%)
	Daily	2 (0.8%)	80 (4.6%)
Disturbance of work**	Never	164 (66.9%)	620 (36.0%)
	Sometimes	59 (24.1%)	774 (45.0%)
	Often	20 (8.2%)	279 (16.2%)
	Daily	2 (0.8%)	48 (2.8%)

Cont. + According to GERD Impact Scale.

*p < 0.05, **p < 0.001 (troublesome versus non-troublesome symptoms; chi-squared test).

**Table 2:** Factors associated with suffering from troublesome symptoms among patients off PPI

**Factor**	**Adjusted odds ratio**	**95% CI**	***P*-value**
Hypercholesterolaemia			0.03
No			
Yes	1.57	(1.05; 2.34)	
Acid regurgitation			<0.0001
Never			
Sometimes	2.24	(1.54; 3.26)	
Often	5.35	(3.24; 8.85)	
Daily	8.30	(2.71; 25.42)	
Epigastric burning/pain			0.003
Never			
Sometimes	1.12	(0.76; 1.66)	
Often	2.15	(1.29; 3.59)	
Daily	4.35	(1.29; 14.73)	
Sleep disturbance			<0.0001
Never			
Sometimes	2.41	(1.73; 3.36)	
Often	3.15	(1.84; 5.42)	
Daily	-	(0; 0)	

**Figure 1:**
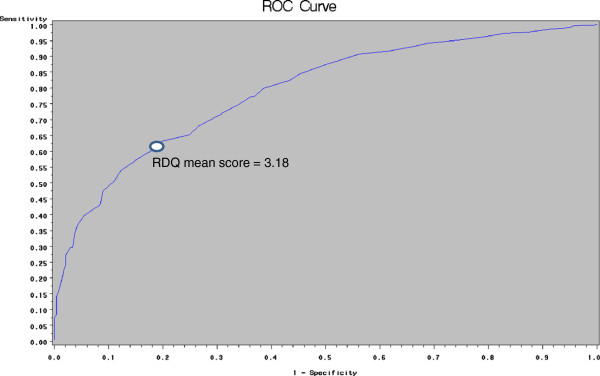
ROC curves for identification of troublesome symptoms with the reflux disease questionnaire in patients off proton pump inhibitor therapy.

Patients without PPI treatment with TS registered a significantly higher RDQ mean score than did those with NTS (3.54 ± 0.94 vs. 2.55 ± 0.77; *p* = <0.0001). Figure 1 depicts the ROC curves of the global RDQ score for identification of TS. The area under the ROC (AUC) was 0.79 for the mean RDQ score. The cut-point of the global RDQ score which maximised Younden's index was 3.18, which yielded a sensitivity of 63.2% and a specificity of 80.2% for identifying TS, and positive and negative predictive values of 96% and 24% respectively.

### Patients on PPI

2606 (57%) of the 4574 patients included were receiving treatment with PPIs, and of these, 2367 (90.8%) reported their symptoms as troublesome. Table 3 shows the clinical characteristics and symptoms of the two groups. Among the patients who reported their symptoms as TS versus NTS, there was a slightly lower proportion of women, a higher proportion of smokers, and a lower proportion who consumed no alcohol or consumed it only occasionally. Likewise, there were differences in symptom frequency between the two groups. As shown in Table 4, factors associated with reporting TS were frequency of acid regurgitation, epigastric pain/burning (probably reflecting related dyspepsia), and sleep disturbance due to reflux symptoms.

**Table 3:** **Characteristics of patients on PPI**, **stratified according to whether symptoms were non**-**troublesome or troublesome**

	**Variable**	**Non**-**troublesome symptoms (N = 239)**	**Troublesome symptoms (N = 2367)**
Age*	Mean ± SD	56.5 ± 14.5	55.0 ± 13.2
Gender*	% females	113 (47.3%)	718 (41.7%)*
Body mass index	Mean ± SD	27.2 ± 3.5	27.1 ± 3.8
Alcohol*	Any	121 (50.6%)	1045 (60.7%)*
Smoking	Current	97 (40.5%)	888 (51.6%)
	Former	95 (39.7%)	520 (30.2%)*
Concomitant therapy	Calcium blockers	25 (10.5%)	186 (10.8%)
	Nitrates	6 (2.5%)	53 (3.1%)
	NSAIDs	28 (11.7%)	201 (11.7%)
	ASA	13 (5.4%)	96 (5.6%)
	Benzodiazepines	44 (18.3%)	367 (21.3%)
Comorbidity	Diabetes	26 (10.8%)	157 (9.1%)
	HBP	86 (36.0%)	608 (35.3%)
	Hypercholesterolemia	60 (25.1%)	442 (25.7%)
	Depression	32 (13.4%)	184 (10.7%)
	Anxiety disorder	52 (21.8%)	434 (25.2%)
	Asthma**	16 (6.7%)	55 (3.2%)
Frequency of symptoms+			
Heartburn	Never	69 (28.9%)	211 (8.9%)
	Sometimes	108 (45.2%)	1036 (43.8%)
	Often	53 (22.2%)	854 (36.1%)
	Daily	9 (3.8%)	265 (11.2%)
Acid regurgitation	Never	72 (30.1%)	212 (9.0%)
	Sometimes	120 (50.2%)	1008 (42.9%)
	Often	38 (15.9%)	880 (37.5%)
	Daily	9 (3.8%)	248 (10.6%)
Chest pain	Never	112 (46.9%)	565 (23.9%)
	Sometimes	89 (37.2%)	1054 (44.5%)
	Often	29 (12.1%)	614 (26.0%)
	Daily	9 (3.8%)	133 (5.6%)
Epigastric burning/pain	Never	58 (24.3%)	284 (12.0%)
	Sometimes	121 (50.6%)	984 (41.6%)
	Often	49 (20.5%)	872 (36.8%)
	Daily	11 (4.6%)	227 (9.6%)
Hoarseness	Never	131 (55.5%)	784 (33.3%)
	Sometimes	70 (29.7%)	1003 (42.6%)
	Often	24 (10.2%)	473 (20.1%)
	Daily	11 (4.7%)	97 (4.1%)
Impact of symptoms+			
Sleep disturbance**	Never	96 (40.2%)	385 (16.3%)
	Sometimes	99 (41.4%)	1213 (51.3%)
	Often	39 (16.3%)	666 (28.2%)
	Daily	5 (2.1%)	100 (4.2%)
Eating disturbance**	Never	87 (36.4%)	396 (16.8%)
	Sometimes	107 (44.8%)	1079 (45.6%)
	Often	37 (15.5%)	756 (32.0%)
	Daily	8 (3.3%)	133 (5.6%)
Disturbance of work**	Never	135 (56.5%)	801 (33.9%)
	Sometimes	78 (32.6%)	1062 (44.9%)
	Often	21 (8.8%)	440 (18.6%)
	Daily	5 (2.1%)	61 (2.6%)

Cont. + According to GERD Impact Scale.

*p < 0.05, **p < 0.001 (troublesome versus non-troublesome symptoms; chi-squared test).

**Table 4:** Factors associated with suffering from troublesome symptoms among patients on PPI

**Factor**	**Adjusted odds ratio**	**95% CI**	***P-value***
Heartburn			0.00
Never			
Sometimes	2.02	(1.39; 2.93)	
Often	2.23	(1.4; 3.54)	
Daily	3.74	(1.63; 8.61)	
Acid regurgitation			<0.0001
Never			
Sometimes	1.84	(1.27; 2.66)	
Often	4.19	(2.56; 6.86)	
Daily	4.16	(1.84; 9.39)	
Sleeping disturbance			0.03
Never			
Sometimes	1.67	(1.18; 2.36)	
Often	1.59	(1; 2.54)	
Daily	1.25	(0.45; 3.49)	

**Figure 2:**
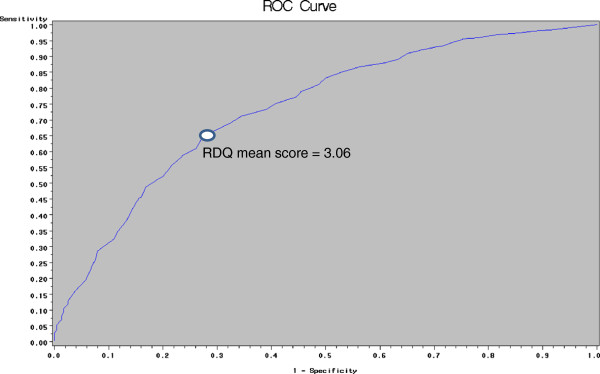
ROC curves for identification of troublesome symptoms with the reflux disease questionnaire in patients receiving proton pump inhibitor therapy.

Figure 2 depicts the ROC curves of the global RDQ score for identification of TS in patients on PPI. The AUC was 0.74 for the global RDQ score. The cut-point of the mean RDQ score which maximised Younden's index was 3.06, which yielded a sensitivity of 65.4% and specificity of 71.8% for identifying TS, and positive and negative predictive values of 96% and 17% respectively.

#### *Statistical power*

Since the target sample size was not attained and the number of unassessable patients was higher than expected, we calculated the study's statistical power. In the case of untreated patients, working with a sample of 1968 patients afforded us a maximum admissible error of δ1 = ±2.28 percentage units for sensitivity (70%) and δ2 = ±4.4 percentage units for specificity (70%).

In the case of treated patients, working with a sample of 2606 patients afforded us a maximum admissible error of 1 = ±1.98% for sensitivity (70%) and δ2 = ±3.82% for specificity (70%).

#### *Severity cut-points*

The sample was analysed jointly to determine these cut-points, in view of the fact that between patients on PPI and patients off PPI there were no relevant differences in predictive factors of TS, nor substantial differences in the optimal cut-point of the RDQ scale for identifying TS in both groups of patients.

Of the 4565 patients who had all the requisite data for being analysed, we classified 1237 (27.1% [25.8%,28.4%]) as mild, 2313 (50.7% [49.2%,52.1%]) as moderate and 1015 (22.2% [21.0%,23.4%]) as severe, on the basis of the reflux dimension of GSRS.

**Figure 3:**
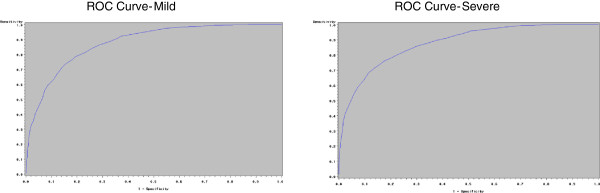
ROC curves for identifying severity of symptoms with the reflux disease questionnaire.

Figure 3 shows the ROC curves for identification of mild and severe symptoms obtained for the RDQ. The cut-points which maximised Younden's index were 2.8 and 3.9, with a sensitivity and specificity of 78.6% and 74.2% respectively for mild symptoms, and 77.3% and 72.1% respectively for severe symptoms.

Simple percentage agreement between the GSRS classification and physicians' subjective opinions was only 59.5%, with a weighted Kappa index of 0.38 (95% CI:(0.36, 0.40). Physician-based classification also showed poor agreement with classifications based on the RDQ (0.36; 95% CI [0.34; 0.38]).

**Figure 4:**
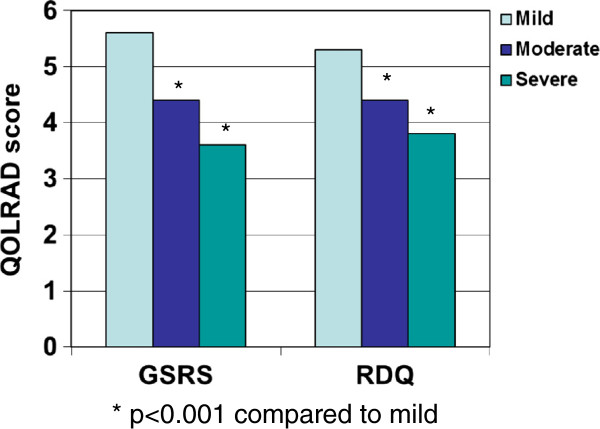
Quality of life (Quality of Life in Reflux and Dyspepsia [QOLRAD] mean score) according to the classification of GERD severity obtained with the Gastrointestinal Symptom Rating Scale (GSRS), and the Reflux Disease Questionnaire (RDQ); * ***p* < 0.001 severe/moderate versus mild.**

Classification of disease severity measured with the GSRS and RDQ corresponded to deterioration in health-related quality of life as measured by the QOLRAD (Figure 4).

## Discussion

Our study's principal contribution is that patients' perception of reflux symptoms as "troublesome" depends, above all, on the characteristics of the symptoms themselves, and that the RDQ is a relatively useful instrument for identifying symptoms regarded as troublesome by the patient. Accordingly, the RDQ would be useful for differentiating troublesome from non-troublesome reflux symptoms (the basis of the Montreal Consensus), thereby adding value to its recently shown ability to make a symptom-based diagnosis of GERD among patients with troublesome upper GI symptoms [16].

The Montreal Consensus [5] lays down a disease definition based on a situation where the symptoms are perceived as troublesome by the patient and the definition's application to clinical practice is feasible [23]. Its implementation depends basically on the physician judging the impact of the symptoms relayed to him by the patient as being or not being troublesome. It is reasonable to surmise that questions about symptom frequency, severity and impact on the patient would have been incorporated into most physicians' anamnesis years ago, and yet there continues to be discordance between physicians' and patients' respective subjective impressions, with disease severity being generally underestimated [11,12,24]. The use of patient-administered questionnaires can thus help physicians ascertain the patients' symptoms more objectively and decide whether such symptoms are indeed troublesome.

The principal aim of this study was to assess the capacity of a simple scale that is widely used in clinical practice and clinical studies -the RDQ- to discern typically troublesome symptoms. In our study, the RDQ displayed good sensitivity and specificity when it came to distinguishing troublesome from non-troublesome symptoms. This is so in the two principal clinical situations provoked by patients with typical GERD symptoms, namely, the need to decide whether the symptoms are constitutive of disease (GERD diagnosis); and the persistence of symptoms with treatment (adequate or inadequate response). The negative predictive values are low because they depend on prevalence, and in this case, the prevalence of troublesome symptoms is so high that it increases the amount of false negatives.

Furthermore, the cut-points for identifying troublesome symptoms in the RDQ are homogeneous across all patients, with or without PPI treatment. Patients' perception of TS appears to be fairly uniform and homogeneous over the disease course, something that simplifies clinical application considerably, since the same result is obtained in two populations of patients under different circumstances. It seems reasonable to propose that, as GERD is defined on the basis of the presence of troublesome symptoms, inadequate response to treatment should be defined in like manner.

Moreover, other recent studies have shown that RDQ is useful for diagnosis of GERD in real-world practice [25].

The factors that influence the perception of symptoms as troublesome are not well known, though there are undoubtedly subjective elements personal to each patient, and it has been reported that in the general population such perception can depend on factors extraneous to the symptoms themselves [26]. While the aim of our study was not specifically to study this aspect, our data suggest that, among patients who consult primary care physicians, apart from the frequency of typical symptoms, impact on sleep is a relevant characteristic in such symptoms being viewed as troublesome. In this regard, recent studies conducted mainly in Western countries have shown a two-way relationship between GERD and sleep, in cases where night-time reflux leads to sleep deprivation and sleep deprivation *per se* can exacerbate GERD by enhancing perception of intra-oesophageal stimuli [27-29]. Other factors of a socio-cultural nature may have an influence: in our study, we covered all areas country-wide to try and minimise the potential impact that these factors might have. We did not conduct a comprehensive study of all the potential factors that might exert an influence, psychosocial factors in particular. This was not the primary goal of the study and would have required increasing the number of questions to be completed by patients, thereby endangering compliance with the protocol and, by extension, putting the primary goal at risk. Nevertheless, the fact that depression and anxiety disorder diagnosed by the physician may not represent a related factor, suggests that psychosocial factors play a secondary role in the primary care setting. It would seem that neither other comorbidities nor the use of drugs play a relevant role, though it has to be said that patients with severe diseases which might potentially have had a substantial impact on health-related quality of life were excluded. It should be noted that hypercholesterolemia was associated with reporting TS. Although there is no evident explanation, this does not appear to be a random result, given the great difference in prevalence in both groups. The fact that it was not associated with TS among patients on treatment suggests that, in reality, hypercholesterolemia is associated with GERD rather than with troublesome symptoms. In this respect, this association has already been reported by Ponce et al. [30] in this country and was recently observed in a population-based study by Eslick and Talley [31]. It has been pointed out that this association could be mediated by obesity but in our study this relationship persisted after adjusting for BMI. At all events, frequency of symptoms plus their impact on sleep emerge as the principal factors for determining TS.

There are no universally accepted severity scales for GERD, and indeed the use of the GSRS was the only Spanish-language standard available. Obviously, "severity", like "troublesome", is a subjective measure and difficult to define, seeing as many factors can be responsible for it. Nonetheless, the RDQ displayed a great capacity for discerning these groups when the GSRS was used as the standard. This tells us two things: first, that having cut-points enables patients to be stratified, which is undeniably useful in clinical practice and clinical research alike; and second, that the scales indirectly indicate a relationship with severity and so express severity quantitatively, which enables changes in severity to be assessed. It will be for future studies to demonstrate this.

Our results are important because they mean that a therapeutic attitude can be uniformly implemented and treatment escalations can be modulated, and because they provide a single cut-point for cases where PPIs are or are not being taken, which is vital when it comes to defining therapeutic failure for every individual patient.

Our study has a number of strengths. The first is its large sample size. The inclusion of over 4,500 patients allowed us, not only to ensure a sufficient number of persons with NTS, but also to provide statistical power to detect small yet potentially relevant effects. Furthermore, the study population represents clinical reality, in that persons with troublesome and non-troublesome symptoms who consulted physicians were studied, free of any interference in the form of persons with symptoms not seeking medical consultation. Secondly, all the instruments used to collect data were validated, with the exception of the question referring to TS (which solely seeks to represent the clinical question homogeneously), and the clinical information was drawn directly from the data source (the physician). Although the degree to which a given symptom is troublesome can be evaluated numerically, as in the case of a recent population-based study [26], we nevertheless feel that a yes/no question obliges patients to define themselves, prior to any other question on the impact of symptoms, and so conforms to the Montreal philosophy, i.e., that it is for the patient to define what is and what is not troublesome. Thirdly, patients from over 900 health centres were included, thereby avoiding any single-researcher bias vis-à-vis the patient profile included. Fourthly, the inclusion criteria were as lax as possible, so as to enable a truly representative sample of patients with GERD symptoms seeking medical advice to be included. The exclusions were only those required by protocol, as well as that of patients with diseases which might have an important impact on quality of life.

It is also necessary to acknowledge some limitations. Our study was observational and thus the validity of the cut-points was not confirmed in other populations. Even so, it must be stressed that the same cut-points were reproduced in both subsamples, which indirectly indicates the validity of these instruments. No sub-analysis was performed to try and identify the questionnaire items which were most relevant or even whether the combination of the two questionnaires or of the most relevant items from both might have had greater efficacy. The standard used was strictly based on symptoms and specifically on the simple specification by the patient that he/she had suffered from heartburn/acid regurgitation and that this was troublesome. To what extent this is representative of the diagnosis obtained using invasive physiological tests is uncertain. Despite the fact that a recent study reported a low sensitivity for clinical diagnosis based on the Montreal Consensus in obese patients [32], the use of the RDQ for symptom-based diagnosis of GERD in the primary-care setting has been clinically validated [16], and all clinical guidelines support symptom-based diagnosis of GERD.

## Conclusions

In brief, it can be concluded that patients' perception of reflux symptoms as troublesome depends, above all, on the characteristics of the symptoms themselves and that the RDQ in a useful instrument for helping the primary care physician differentiate relevant from irrelevant reflux symptoms, which is fundamental for diagnosis of GERD based on the Montreal Consensus.

## Competing interest

M Muñoz is an employee of AstraZeneca Spain. J Zapardiel was an employee for AstraZeneca Spain at the time of designing the study and collecting and analysing the data.

## Authors’ contributions

ER has had full access to all of the study data and takes responsibility for the integrity of the data and the accuracy of the data-analysis. Study concept and design: ER, JZ, ES, MM, MDR; analysis and interpretation: ER, JZ, ES; drafting the manuscript: E.R, M.B, J.Z, M.D.R; critical review of the manuscript for important intellectual content: E.R, M.B, J.Z, E.S, M.M; statistical analysis: ES. All authors read and approved the final manuscript.

## Pre-publication history

The pre-publication history for this paper can be accessed here:

http://www.biomedcentral.com/1471-230X/14/17/prepub
